# Teachers’ Responses to Cyberbullying: Psychometric Validation and Latent Profile Analysis Among Chinese Teachers

**DOI:** 10.3390/bs16081300

**Published:** 2026-07-31

**Authors:** Meng Sun, Zelin Lu, Jiutong Luo

**Affiliations:** 1 College of Education for the Future, Beijing Normal University, Zhuhai 519087, China; msun@bnu.edu.cn; 2 Faculty of Education, Shenzhen University, Shenzhen 518060, China; 2310487017@email.szu.edu.cn

**Keywords:** teachers’ responses, cyberbullying, scale validation, confirmatory factor analysis, latent profile analysis

## Abstract

Cyberbullying among school-age children has drawn a wide range of attention, and teachers are important in intervening in cyberbullying among students. However, there is still a lack of knowledge regarding teachers’ responses to cyberbullying. Thus, this study aims to validate a scale to measure teachers’ responses to cyberbullying and identify teachers’ profiles with a person-centered approach among Chinese teachers. A total of 486 Chinese teachers (78.4% female, Mean_age_ = 36.80 years, SD = 9.81) completed the questionnaire on a scale adopted from the traditional bullying context. Results of the CFA confirmed the validity of the teachers’ responses to the cyberbullying scale. Latent profile analysis revealed four teacher profiles, i.e., passive, inconsistent, medium, and high, for teachers’ responses to cyberbullying. Further analyses indicated significant differences among the identified subgroups. Teachers’ self-efficacy towards intervening in cyberbullying also confirmed the latent profile results. This study provides implications for teacher professional development in addressing cyberbullying among students.

## 1. Introduction

With the widespread use of the Internet and the increasing integration of personalized digital technologies into adolescents’ everyday lives, online interaction has become a central component of their social development. Social networking platforms, instant messaging applications, and online gaming environments provide adolescents with continuous opportunities for communication and self-expression. However, the expansion of digital communication spaces has also created new contexts for interpersonal aggression, including cyberbullying—a pervasive social problem in the digital age (Juvonen & Gross, 2008; Xu et al., 2024). According to the literature, cyberbullying is generally defined as intentional behavior by an individual or group that causes harm or discomfort to others through digital media, typically involving repeated aggression and a power imbalance in online contexts (Tokunaga, 2010).

Compared with traditional forms of bullying, cyberbullying is often characterized by anonymity, rapid dissemination of harmful content, permanence of digital traces, and the potential involvement of a vast and invisible audience. These features not only make cyberbullying more difficult to monitor and regulate but may also intensify victims’ psychological distress and extend the duration of harm. Moreover, accumulating evidence indicates that cyberbullying has become increasingly prevalent among adolescents worldwide. Empirical studies across cultural contexts report rising rates of both victimization and perpetration over the past decades. For instance, 12.5% of adolescents aged 14–17 reported experiencing cyberbullying within a three-month period in 2015 (Waasdorp & Bradshaw, 2015). More recent data suggest even higher prevalence in certain regions. A large-scale study in China found that 37.5% of middle school students reported involvement in cyberbullying, either as perpetrators or victims, and that greater time spent online was associated with more complex and multi-faceted cyberbullying experiences (J. Li et al., 2023).

Beyond prevalence, the consequences of cyberbullying are particularly concerning. Due to the anonymity and invisibility afforded by digital platforms, cyberbullying is often more difficult to detect than traditional bullying (e.g., Peebles, 2014; Vaillancourt et al., 2017), which may delay timely intervention. Research suggests that cyberbullying can result in severe emotional, social, and academic consequences for victims, sometimes exceeding those associated with face-to-face bullying (Waasdorp & Bradshaw, 2015). Moreover, perpetrators themselves are not immune to negative outcomes; they have been found to exhibit higher levels of aggressive behavior, social anxiety, and lower self-esteem (Martinez-Monteagudo et al., 2020). Taken together, these findings highlight the urgent need to identify key agents within adolescents’ ecological systems and to develop effective strategies to prevent cyberbullying and mitigate its adverse consequences.

In this study, we focus specifically on the role of teachers in addressing cyberbullying. The importance of teachers has been well established in the traditional bullying literature (e.g., Campaert et al., 2017; Gest & Rodkin, 2011) and is increasingly supported by research directly examining cyberbullying (Guarini et al., 2019; Nagar & Talwar, 2023). In traditional bullying contexts, teachers’ intervention strategies have been shown to play a pivotal role in shaping classroom norms and peer dynamics. For example, Gest and Rodkin (2011) emphasized the necessity of structured anti-bullying practices implemented by teachers, while Campaert et al. (2017) demonstrated that teachers’ active responses were associated with reductions in bullying behaviors, whereas passive or non-responses were linked to higher levels of bullying. Emerging evidence in cyberbullying contexts echoes these findings. Guarini et al. (2019) showed that teachers can enhance students’ awareness of cyberbullying and promote more coping strategies. Similarly, Nagar and Talwar (2023) found that supportive teacher strategies significantly increased adolescents’ willingness to disclose cyberbullying experiences, compared to situations in which teachers provided little or no support. These findings collectively suggest that teachers are critical agents in cyberbullying prevention and intervention, underscoring the importance of systematically examining how teachers respond to cyberbullying situations.

However, when examining teachers’ roles in cyberbullying, at least two critical research gaps remain to be addressed. On the one hand, no valid scale measures teachers’ responses to cyberbullying. Although it is possible to address this issue by simply adopting the existing scale in the traditional bullying context (Campaert et al., 2017; Nappa et al., 2021), a statistical validation process is needed. In particular, confirmatory factor analysis (CFA) should be performed to ensure that models are theoretically sound and empirically valid. On the other hand, existing studies mainly utilize the survey method with variable-centered analytic approaches, such as multiple regression and structural equation modeling, to investigate the cyberbullying issue among teachers. Although these approaches provide valuable insights into the relationships among variables, they assume that the population is relatively homogeneous and therefore may overlook meaningful heterogeneity in teachers’ response patterns. In practice, teachers may exhibit different combinations of responses to cyberbullying (e.g., high victim support but low disciplinary approaches), even when they have similar overall levels of responsiveness. A person-centered approach, such as latent profile analysis (LPA; Howard & Hoffman, 2018), can identify subgroups of teachers who share similar patterns across multiple response strategies, thereby providing a more nuanced understanding of teachers’ behavioral tendencies and informing more targeted professional development (Luo et al., 2025). In the traditional bullying context, a recent study conducted with the person-centered approach categorized teachers’ responses to bullying into three profiles: highly active, moderately active, and passive (van Gils et al., 2024), and implies that teachers could reduce bullying by taking various active responses. Building on this work, the present study extends LPA to the cyberbullying context to examine whether distinct response profiles also exist among teachers facing cyberbullying situations.

### 1.1. Teachers’ Responses to Cyberbullying

Recent researchers have distinguished teachers’ responses to traditional bullying into five categories: non-intervention, disciplinary method(s), mediation, group discussion, and victim support (Campaert et al., 2017; Nappa et al., 2021). Although cyberbullying differs from traditional bullying in several important respects, including anonymity, digital permanence, and reduced teacher visibility (e.g., Macaulay et al., 2018), both forms involve intentional peer aggression that requires teachers to make decisions regarding intervention, discipline, conflict resolution, and victim support. Therefore, while the specific implementation of these responses may vary across contexts, the underlying response strategies remain conceptually applicable to cyberbullying. Following this rationale, it is possible to adapt these five-dimensional concepts to the context of cyberbullying. The detailed explanations for these five responses to cyberbullying are elaborated below.

Non-intervention represents a teacher who does not intervene in a cyberbullying situation, either intentionally or because the teacher is not aware of cyberbullying (Nappa et al., 2021; van Gils et al., 2022; van Gils et al., 2024). It is not surprising that most teachers do not suitably deal with cyberbullying (DeSmet et al., 2015). In traditional school bullying, some teachers may consider bullying as normative behavior (Kochenderfer-Ladd & Pelletier, 2008). Additionally, some teachers may also consider the specific type of bullying less problematic, which is often the case for reasonable bullying (Blain-Arcaro et al., 2012). Such beliefs may also exist in the cyberbullying context and lead to non-intervention responses among teachers.

Disciplinary methods imply that a teacher sanctions student(s) who bullied others (Nappa et al., 2021; van Gils et al., 2022; van Gils et al., 2024). When teachers punish perpetrators of bullying, they condemn the behavior and thus focus on the action (Campaert et al., 2017). Disciplinary methods are teachers’ positive responses, which may message the perpetrators that their behaviors are forbidden or illegal and motivate the stakeholders or bystanders to intervene with the victims (Wachs et al., 2019). Such a response may also be effective in the cyberbullying context.

Group discussion means a teacher involves the whole class or a group of students to discuss cyberbullying situations (Nappa et al., 2021; van Gils et al., 2022; van Gils et al., 2024). Group discussion is distinguished as a response to victimization (Colpin et al., 2021), which is neither positive nor negative. However, group discussions are relatively more straightforward to carry out among students and are an effective way to raise students’ awareness of cyberbullying. By participating in the discussion, students gain various perspectives on cyberbullying from other students. In particular, cyberbullies could learn the thoughts of victims, thus reducing cyberbullying behavior. Group discussion is also one of the most common teachers’ responses to bullying immediately following the incident, aiming at helping the students to solve the problem (Colpin et al., 2021). Thus, group discussion may be an effective response to stop cyberbullying.

Mediation implies that teachers act informally as an intermediary to allow the involved students to express their points of view (Nappa et al., 2021; van Gils et al., 2022; van Gils et al., 2024). Mediation is thought to be a kind of restorative intervention and is suggested to be more effective than punitive ones (e.g., Garandeau et al., 2016). Mediation by teachers is a multi-faceted approach because it not only addresses the immediate conflict but also fosters an environment of respect and understanding among students. Thus, mediation may be an effective response to stop cyberbullying.

Victim support refers to teachers providing direct support to students who have experienced cyberbullying (Nappa et al., 2021; van Gils et al., 2022; van Gils et al., 2024) and specifically targets the immediate needs and well-being of the victim. Research also underlines that intervention strategies, such as victim support, are more effective than punitive ones (e.g., Garandeau et al., 2016). Wachs et al. (2019) also suggested that students considered teachers’ supportive and relational intervention strategies particularly effective in preventing cyberbullying. In this case, victim support may be an effective response to stop cyberbullying. It has been proven to be a more effective way of intervention in cyberbullying than punitive ways (Garandeau et al., 2016).

According to a study in a traditional bullying context (van Gils et al., 2024), teachers may not take only one response but use a variety of intervention strategies. Thus, analyzing the teachers’ profiles according to their responses to cyberbullying is essential.

### 1.2. Latent Profile Analysis

Latent profile analysis (LPA) is a method for latent variable modeling (Wang & Hanges, 2011), which assumes multiple sub-populations exist within a sample, each characterized by distinct profiles of variable means or probabilities (Spurk et al., 2020). The LPA method assumes individuals within each group have similar patterns in their data, and identifies hidden groups (or classes) that help explain the relationships among the observed continuous variables (Lazarsfeld & Henry, 1968). The LPA method departs from traditional variable-centered approaches, providing a nuanced way to understand a larger population using a specific set of variables as the basis for the de-complexity of individual differences within a population.

The LPA method has been used in bullying and cyberbullying contexts and produces meaningful results. For example, Antoniadou et al. (2019) explored the roles of high school students in incidents of cyber and traditional bullying/victimization and examined the psychosocial and emotional profiles of students categorized into each participant role. They confirmed cyber aggression as a component of a traditional bullying/victimization pattern, and suggested that students should be best categorized according to their behavior rather than the context in which it occurs (Antoniadou et al., 2019). Regarding the teachers, van Gils et al. (2024) revealed that among the identified three teachers’ profiles, two (i.e., highly active and passive) teachers’ response profiles were related to reduced self-reported bullying among students. However, most existing research about teachers’ responses to cyberbullying uses a variable-centered approach to clarify how key variables of interest are co-related within a population (Howard & Hoffman, 2018). However, the variable-centered approach makes it hard to determine if subgroups of similar subjects exist within a given group (Howard & Hoffman, 2018). Given the increase in cyberbullying and its seriousness among adolescent students, it is necessary to figure out the classification of teachers’ responses to cyberbullying, which is crucial to intervene and reduce cyberbullying in the school context.

### 1.3. The Present Study

The research aim of this study is twofold. First, this study aims to validate the teachers’ responses to cyberbullying scale in line with the traditional five-dimensional teachers’ responses to bullying scale. Second, this study aims to examine the profiles of teachers’ responses to cyberbullying based on the five-dimensional responses (i.e., non-intervention, disciplinary method, mediation, group discussion, and victim support). Moreover, based on the LPA result, we investigated teachers’ self-efficacy differences to determine whether this factor differed across teachers’ profiles. According to Q. Li (2009), teachers’ self-efficacy has consistently been identified as an important factor associated with teachers’ willingness and confidence to intervene in bullying and cyberbullying situations. Therefore, examining whether the identified response profiles differ in teachers’ self-efficacy may provide additional evidence for the meaningfulness of the latent profiles.

## 2. Methods

### 2.1. Participants

The convenience sampling method was adopted in this study. Teachers from a district in southern China were approached via the local educational association on school moral education. A total of 486 teachers (78.4% female, Mean_age_ = 36.80 years, SD = 9.81) voluntarily participated and finished the questionnaire. Among them, 255 teachers (52.5%) taught in elementary schools and 231 teachers (47.5%) taught in secondary schools. Regarding teaching experience, 220 teachers (45.2%) had less than 10 years, 131 teachers (27.0%) had 10–20 years, and 135 teachers (27.8%) had more than 20 years of teaching experience. The questionnaire was distributed online and took approximately 15 min to complete. This study was conducted in line with the ethics requirements of the authors’ institute. Written consent was obtained from all teachers prior to filling out the questionnaire.

### 2.2. Measures

**Teachers’ responses to cyberbullying:** Based on the scale for teachers’ responses to traditional bullying in the studies of Campaert et al. (2017) and Nappa et al. (2021), we adopted the scale from van Gils et al.’s (2022) work. The adaptation primarily involved modifying item wording to reflect the cyberbullying context while retaining the original conceptual structure and five response dimensions. Specifically, references to bullying were replaced with cyberbullying, and references to victims or bullies were specified as cyber victims or cyberbullies, where appropriate. No substantive changes were made to the underlying constructs or item content. Then, the English version was translated into Chinese and then back-translated by two bilingual researchers to ensure linguistic equivalence. The translated items were subsequently reviewed by an expert in educational psychology and cyberbullying research. Finally, teachers’ responses were evaluated across five dimensions, encompassing fifteen specific items. Participants rated all items on a 5-point Likert scale (1 = Never; 5 = Always). The Cronbach’s alpha(s) are shown in Table 1.

**Teachers’ self-efficacy towards intervening in cyberbullying:** To evaluate teachers’ self-efficacy towards intervening in cyberbullying, we adopted two items from Q. Li’s (2009) work, i.e., “I am confident in identifying cyberbullying” and “I am confident in managing cyberbullying”. Teachers answered inquiries on a 5-point Likert scale (1 = Strongly disagree; 5 = Strongly agree). Cronbach’s alpha for this scale was 0.71 in the current study.

### 2.3. Data Analysis

The data analyses consisted of four steps. First, the confirmatory factor analysis (CFA) was performed with AMOS 24.0 to verify whether the five-dimensional solution fit the data well. In addition to ensuring that all factor loadings were statistically significant and fairly above 0.45 (Comrey & Lee, 1992), we evaluated model fit based on several criteria. Specifically, the χ^2^/df ratio was expected to fall between 1 and 3, the Comparative Fit Index (CFI) and Tucker–Lewis Index (TLI) were expected to exceed 0.90, and the Root Mean Square Error of Approximation (RMSEA) and Standardized Root Mean Squared Residual (SRMR) were both expected to be below 0.08 (Browne & Cudeck, 1993; Hu & Bentler, 1999). Then, Cronbach’s alpha coefficients and descriptive statistics of each scale were calculated using SPSS 26.0. Correlational analysis was also conducted to examine relationships between explanatory and outcome variables. Second, latent profile analysis was performed with Mplus 8.0 to identify profiles of teachers’ responses to cyberbullying. To determine the fit of various models and the number of profiles, we inspected several indices; for example, relative fit information criteria were used to decide a better fit, which consists of Akaike information criterion (AIC), Bayesian information criterion (BIC), and the sample size-adjusted BIC (aBIC). The lower values of these three indices indicate a better fit. Meanwhile, the higher the entropy value for different classification models, the better the classification accuracy. The likelihood ratio test (LRT) was conducted to find the better fit between k-profiles and the k-1 one. Additionally, the minimum sample within a subgroup was recommended to be above 1% of the total sample (Spurk et al., 2020). Third, teachers’ self-efficacy in intervening in cyberbullying was also analyzed by one-way ANOVA.

## 3. Results

### 3.1. The Validation of the Teachers’ Responses to Cyberbullying Scale

The initial model with 15 items acceptably fit the data, i.e., χ^2^/df = 3.175, *p* < 0.001, CFI = 0.962, TLI = 0.948, RMSEA = 0.067 (90% CI [0.058, 0.076], SRMR = 0.048). The means and SDs for each item, standardized factor loadings, and Cronbach’s alphas are shown in Table 1. The Cronbach’s alphas for non-intervention, mediation, group discussion, victim support, and disciplinary method were 0.71, 0.70, 0.92, 0.90, and 0.76, respectively.

### 3.2. Descriptive Statistics and Correlational Analysis

Table 2 shows the descriptive statistics and the correlational analysis results. According to the results, non-intervention is negatively related to the other four responses, i.e., mediation, group discussion, victim support, and disciplinary method. Mediation, group discussion, victim support, and disciplinary method are all positively correlated with each other, ranging from 0.51 to 0.72. Teachers’ self-efficacy is negatively correlated with non-intervention and positively related to mediation, group discussion, victim support, and disciplinary method.

### 3.3. Profiles of Teachers’ Responses to Cyberbullying

Models with one to five profiles were compared to figure out the number of profiles based on teachers’ five responses to cyberbullying. Table 3 presents the values for the evaluation of models. The selection of the optimal model was based on multiple criteria, including AIC, BIC, adjusted BIC, entropy, LMR test, and the interpretability of the profiles. Although the five-profile model showed slightly lower AIC, BIC, and adjusted BIC values, it demonstrated lower entropy and a less significant LMR test compared with the four-profile model. Moreover, the additional profile identified in the five-profile solution did not provide substantial theoretical advantages over the four-profile solution. Thus, the 4-profile model was the best fit after comparison.

The results showed unsatisfactory levels of all five responses in profile 1, which was higher for the non-intervention and lower for the other four intervention approaches (i.e., mediation, disciplinary method, group discussion, and victim support). Thus, profile 1 was labeled “passive.” Profile 2 has almost the same level of non-intervention as profile 1, and the levels of other responses in this profile were all higher than those in profile 1. Especially, the value for active responses (i.e., mediation, group discussion, victim support, and disciplinary method) ranged from 2.8 to 3.01. In this case, profile 2 was labeled “Inconsistent”. However, profile 3 and profile 4 significantly differed from the previous two profiles, whose non-intervention levels were lower, and the other four kinds of response levels were also higher. Moreover, comparing profile 3 and profile 4, profile 4 has less non-intervention and more significant intervention characteristics than profile 3. Thus, profile 3 was labeled as “Medium” and profile 4 was labeled as “High”.

**Figure 1:**
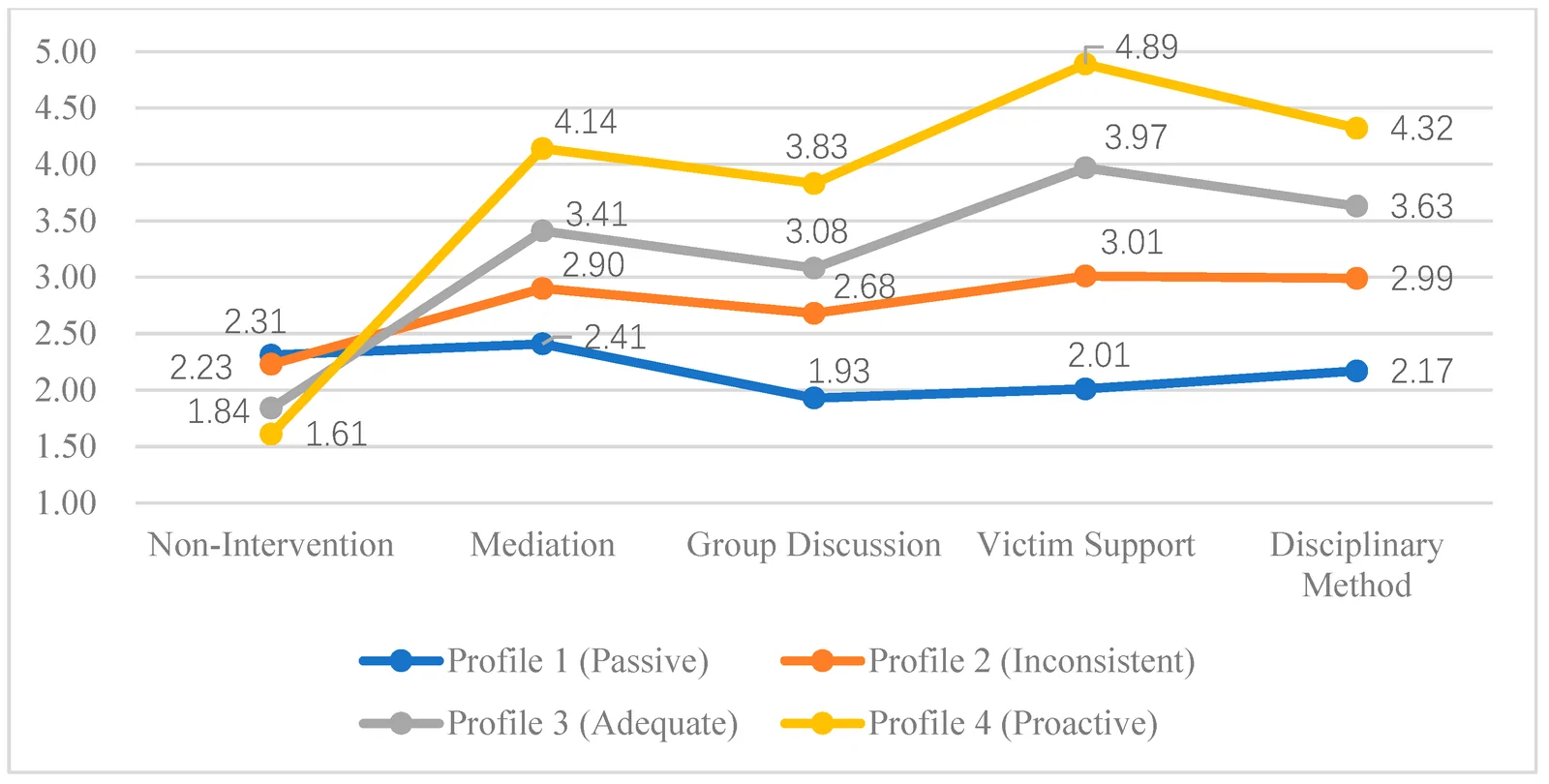
Profiles for teachers’ responses to cyberbullying.

Furthermore, one-way ANOVAs were conducted to compare the differences in Mean (SD) of four profiles across five responses, and results were presented in Table 4. The post hoc multiple comparisons with adjusted *p*-values found significant differences. For non-intervention (F(3, 482) = 19.236, *p* < 0.001), means of profile 1 (Mean = 2.31, SD = 0.64) and profile 2 (Mean = 2.23, SD = 0.77) were equal, while both of them significantly higher than profile 3 (Mean = 1.84, SD = 0.73) and 4 (Mean = 1.61, SD = 0.72). Meanwhile, profile 3 was also higher than profile 4 in non-intervention. For mediation [F(3, 482) = 120.205, *p* < 0.001], group discussion [F(3, 482) = 80.855, *p* < 0.001], victim support [F(3, 482) = 2994.637, *p* < 0.001], and disciplinary method [F(3, 482) = 182.413, *p* < 0.001], the means across four profiles showed similar patterns with the value increased sequentially (i.e., profile 1 < 2 < 3 < 4). Figure 1 shows the profiles’ characteristics across five subdimensions.

### 3.4. Differences in Teachers’ Self-Efficacy Towards Intervening in Cyberbullying Across Four Profiles

To further explore the significance of the four profiles, we used teachers’ self-efficacy in intervening in cyberbullying as the reference. According to the one-way ANOVA, teachers’ self-efficacy towards intervening in cyberbullying was found to be significantly related to teachers’ profiles [F(3, 482) = 46.329, *p* < 0.001]. As shown in Table 5, the Means of profile 1 (Mean = 3.07, SD = 0.37) and profile 2 (Mean = 3.05, SD = 0.44) were equal, while both were significantly lower than profile 3 (Mean = 3.42, SD = 0.49) and profile 4 (Mean = 3.77, SD = 0.66). The self-efficacy of profile 4 was also higher than that of profile 3.

## 4. Discussion

To the best of our knowledge, the study is among the very few studies to validate teachers’ responses to cyberbullying and to investigate the profiles of teachers based on their responses. While most existing studies used the variable-centered approach, this study addressed a very significant literature gap in categories of teachers’ responses to cyberbullying with latent profile analysis (LPA) and identified four subgroups of teachers, i.e., passive, inconsistent, medium, and high. The results also improved our understanding of teachers’ efficacy levels to intervene in cyberbullying.

### 4.1. The Five-Dimensional Teachers’ Responses to Cyberbullying Scale

The teachers’ responses to bullying scale was adapted to the cyberbullying context in this study (Campaert et al., 2017; Nappa et al., 2021; van Gils et al., 2022). The original scale was developed for students and contained five subscales corresponding to the teacher’s five responses (non-intervention, mediation, disciplinary approach, group discussion, and victim support). Each subscale contained three items. To accommodate our study, we altered the original scale to make it applicable to the role of the teacher and conducted a confirmatory factor analysis (CFA) to validate the scale’s factor structure. The results confirmed a 15-item scale of teachers’ responses to cyberbullying (see Appendix A). The findings contribute to the literature in two aspects. On the one hand, it adapted the scale from a student evaluation to teachers’ perceptions, which might be useful in future teachers’ studies or interventions to improve their active responses. On the other hand, the validated five-dimensional scale of teachers’ responses to cyberbullying might further enable the comparison between teachers’ responses to traditional bullying and cyberbullying in the future.

### 4.2. Four Profiles of Teachers’ Responses to Cyberbullying

Teachers were categorized into four different profiles based on their responses to cyberbullying with latent profile analysis (LPA). The results supported the use of LPA in teachers’ responses to cyberbullying. The passive group (profile 1), the smallest category with 6.2% of the sample, is characterized by a higher level of non-intervention and lower levels of mediation, group discussion, victim support, and disciplinary methods. The inconsistent group (profile 2), which accounts for 30.5% of the sample, is characterized by a higher level of non-intervention than the passive group, but is also moderate in the other four active responses to cyberbullying. The medium group (profile 3) accounts for the largest proportion (i.e., 38.5%) of the sample, with lower non-intervention than the passive and inconsistent group, but higher active responses to cyberbullying than the inconsistent and passive groups. Last but not least, the high group, which accounts for about 24.9% of the sample, was even higher than the medium group on mediation, group discussion, victim support, and disciplinary method. The results on teachers’ self-efficacy towards intervening in cyberbullying also supported the four-profile solution. The passive and inconsistent groups were similar and had the lowest levels of self-efficacy towards intervening in cyberbullying, while the high group had the highest self-efficacy, and the medium group was slightly lower. The results confirmed that teachers in the medium and high groups reported higher levels of self-efficacy toward intervening in cyberbullying incidents, suggesting greater perceived readiness and confidence to intervene. Future research is needed to investigate whether these differences are reflected in actual intervention behaviors.

In searching for the existing research, only van Gils et al. (2024) used LPA to identify teachers’ profiles in the traditional bullying context. Our research, partially in line with their three-profile solution (i.e., passive, moderately active, and highly active; van Gils et al., 2024), extended LPA to the cyberbullying context and produced four profiles (i.e., passive, inconsistent, medium, and high). For example, the passive profile is the smallest in both studies. The largest profile in van Gils et al. (2024) was the highly active profile, while the largest profile in this study was the medium profile. While van Gils et al. (2024) adopted the students’ reported data, we collected data from teachers directly in this investigation. To the best of our knowledge, very few studies have applied LPA to teachers’ self-reported data within the context of cyberbullying. Thus, our study provides a more nuanced understanding of the different types of teachers involved in cyberbullying intervention, offering preliminary insights that may help educators tailor professional development for teachers with different response patterns. In particular, teachers in the passive and inconsistent profiles may benefit from additional professional development focusing on mediation, group discussion, victim support, and disciplinary approaches.

Meanwhile, our study also indicated that teachers were often capable of multiple active responses. However, in practical settings, their responses might be biased. For example, Macaulay et al. (2018) showed that teachers might focus on disciplining cyberbullies rather than supporting victims, leading to uneven interventions that may fail to comprehensively address the issue. These findings suggest that encouraging teachers to consider multiple response strategies may be beneficial when addressing cyberbullying incidents. However, these findings should not be interpreted as evidence that the identified profiles directly reflect teachers’ actual intervention behaviors. Future research incorporating behavioral measures or observational data is needed to further validate the practical implications of the identified profiles.

### 4.3. Limitations and Future Research Directions

However, this research also has some limitations. First, the sample was obtained through convenience sampling from one district in southern China and may not be representative of teachers across different regions of China or other cultural and educational contexts. Therefore, the findings should be generalized with caution beyond the present sample. Second, we relied solely on teachers’ self-reported responses, which may be influenced by social desirability bias, as teachers may be inclined to report more active and supportive responses to cyberbullying than they would demonstrate in practice. Consequently, the findings may not fully reflect teachers’ actual intervention behaviors. Moreover, potentially relevant contextual variables, such as teachers’ digital competence, were not assessed in the present study. Future research should recruit more representative samples from diverse regions and cultural contexts, incorporate student-reported or multi-informant data, and examine teachers’ actual intervention behaviors in cyberbullying situations. Third, although the present study provided evidence for the factorial validity and internal consistency of the adapted scale, other psychometric properties, such as measurement invariance, convergent validity, discriminant validity, and test–retest reliability, were not examined. Future research should further evaluate these properties to establish the robustness and generalizability of the instrument across different teacher populations and over time. Fourth, the self-efficacy measure used in this study consisted of only two items. Although the scale demonstrated acceptable internal consistency, the limited number of items may not fully capture the complexity of teachers’ self-efficacy beliefs. Therefore, future studies should employ more comprehensive measures to further examine the role of self-efficacy in shaping teachers’ responses to cyberbullying. Furthermore, in addition to self-efficacy, future research could further validate the identified profiles by examining their associations with a broader range of theoretically relevant external variables (e.g., previous cyberbullying training, school policy awareness, and intervention behaviors). Finally, the comparison of self-efficacy across profiles was based on the most likely profile membership assigned to each participant. Although this approach is commonly adopted in exploratory LPA studies, it does not fully account for classification uncertainty. Future research could adopt a more rigorous BCH (i.e., the Bolck–Croon–Hagenaars) approach to further examine distal outcomes while considering the probabilistic nature of latent profile membership.

## 5. Conclusions

This study generally validated the teachers’ responses to the cyberbullying scale and identified four teacher profiles based on their responses to cyberbullying. The results showed that most teachers are capable of various responses to cyberbullying. Additionally, different profiles of teachers also have different self-efficacy toward intervening in cyberbullying. In general, it moves beyond the traditional variable-centered approach and examines the potential subgroups beyond a “one-size-fits-all” assumption, promoting more nuanced understandings of teacher behavior in cyberbullying interventions. This study implies that we should design different professional development programs for teachers with different profiles. While improving active responses, including mediation, group discussion, victim support, and disciplinary methods, are important for passive and inconsistent groups, reminding teachers to use a balanced use of intervention strategies, such as providing relevant examples and showing the consequences of unbalanced use of strategies, may also be important for medium and high teachers in their professional development program.

## Figures and Tables

**Table 1:** The CFA results.

Items	Mean	SD	Standardized Factor Loading	Cronbach’s Alpha
Non-Intervention (NI)				0.71
NI01	1.94	1.02	0.76	
NI02	2.14	0.99	0.73	
NI03	1.72	0.92	0.52	
Mediation (ME)				0.70
ME01	3.20	1.08	0.70	
ME02	3.57	0.96	0.49	
ME03	3.35	0.99	0.86	
Group Discussion (GD)				0.92
GD01	3.10	0.97	0.89	
GD02	3.02	1.00	0.86	
GD03	3.09	0.94	0.90	
Victim Support (VS)				0.90
VS01	3.58	0.96	0.93	
VS02	3.86	0.93	0.94	
VS03	3.91	0.93	0.72	
Disciplinary Method (DM)				0.76
DM01	3.95	0.95	0.88	
DM02	3.34	1.01	0.58	
DM03	3.27	1.05	0.57	

**Table 2:** Descriptive statistics and correlations among variables.

Variables	1	2	3	4	5	6
Non-Intervention	—					
Mediation	−0.30 ***	—				
Group Discussion	−0.21 **	0.62 ***	—			
Victim Support	−0.33 ***	0.65 ***	0.57 ***	—		
Disciplinary Method	−0.27 ***	0.53 ***	0.51 **	0.72 ***	—	
Self-efficacy	−0.28 ***	0.39 ***	0.37 ***	0.44 ***	0.44 ***	—
Mean (SD)	1.93 (0.78)	3.38 (0.80)	3.07 (0.90)	3.79 (0.85)	3.52 (0.83)	3.37 (0.59)

Note. ** *p* < 0.01; *** *p* < 0.001.

**Table 3:** The LPA results.

Profiles	k	Log Likelihood	AIC	BIC	aBIC	Entropy	LMR	BLRT	Percentages
Profile 1	10	−2992.558	6005.117	6046.979	6015.239				
Profile 2	16	−2638.692	5303.385	5370.364	5319.581	0.833	<0.0001	<0.0001	0.449\0.551
Profile 3	22	−2550.443	5144.886	5236.983	5167.156	0.848	<0.0001	<0.0001	0.101\0.528\0.370
Profile 4	28	−2418.941	4893.882	5011.096	4922.226	0.976	<0.0001	<0.0001	0.062\0.246\0.387\0.304
Profile 5	34	−2383.298	4834.597	4976.928	4869.014	0.918	0.0333	0.0362	0.063\0.150\0.300\0.245\0.243

Note. AIC = Akaike Information Criterion; BIC = Bayesian Information Criterion; aBIC = adjusted Bayesian Information Criterion; LMR = Lo-Mendell-Rubin likelihood ratio test; BLRT = Bootstrap Likelihood Ratio Test.

**Table 4:** Means, standard deviations, and one-way ANOVA results for four profiles of teachers’ responses to cyberbullying.

Dimensions	Profile 1 (Passive)	Profile 2 (Inconsistent)	Profile 3 (Medium)	Profile 4 (High)	One-Way ANOVAs		
	N = 30 (6.2%)	N = 148 (30.5%)	N = 187 (38.5%)	N = 121 (24.9%)			
	Mean (SD)	Mean (SD)	Mean (SD)	Mean (SD)	df	F	Post Hoc
Non-Intervention	2.31 (0.64)	2.23 (0.77)	1.84 (0.73)	1.61 (0.72)	3	19.236 ***	Profile (1 = 2 > 3 > 4)
Mediation	2.41 (0.54)	2.90 (0.49)	3.41 (0.64)	4.14 (0.69)	3	120.205 ***	Profile (1 < 2 < 3 < 4)
Group Discussion	1.93 (0.57)	2.68 (0.60)	3.08 (0.75)	3.83 (0.88)	3	80.855 ***	Profile (1 < 2 < 3 < 4)
Victim Support	2.01 (0.24)	3.01 (0.19)	3.97 (0.20)	4.89 (0.18)	3	2994.637 ***	Profile (1 < 2 < 3 < 4)
Disciplinary Method	2.17 (0.57)	2.99 (0.47)	3.63 (0.57)	4.32 (0.67)	3	182.413 ***	Profile (1 < 2 < 3 < 4)

Note. *** *p* < 0.001; *p*-value was adjusted to 0.017 (i.e., 0.05/3) to evaluate multiple comparisons in the post hoc tests.

**Table 5:** Differences in teachers’ self-efficacy towards intervening in cyberbullying.

Dimensions	Profile 1 (Passive)	Profile 2 (Inconsistent)	Profile 3 (Medium)	Profile 4 (High)	One-Way ANOVAs		
	Mean (SD)	Mean (SD)	Mean (SD)	Mean (SD)	df	F	Post Hoc
Self-efficacy	3.07 (0.37)	3.05 (0.44)	3.42 (0.49)	3.77 (0.66)	3	46.329 ***	Profile (1 = 2 < 3 < 4)

Note. *** *p* < 0.001; *p*-value was adjusted to 0.017 (i.e., 0.05/3) to evaluate multiple comparisons in the post hoc tests.

## Data Availability

Data are available upon request from the authors.

## Appendix A

### Teachers’ Responses to Cyberbullying Scale

Non-Intervention

1. I do nothing about cyberbullying.
2. I do not notice the cyberbullying.
3. I let students solve cyberbullying alone.

Mediation

4. I help students involved to solve the cyberbullying.
5. I try to get the students to make peace online.
6. I help the (involved) students to find a solution for the cyberbullying episode.

Group Discussion

7. I discuss cyberbullying with the whole class
8. I discuss with the whole class how much the victim can suffer as a result of the cyberbullying.
9. I explain what cyberbullying is and discuss it with the class.

Victim Support

10. I try to provide comfort and support to the cyber victim within the classroom.
11. I try to help the cyber victim.
12. I console the cyber victim.

Disciplinary Method

13. I say to the cyberbully/cyberbullies that his/her/their behavior is unacceptable.
14. I take measures against the cyberbully/cyberbullies.
15. I report cyberbullying incidents to the school principal or parents.
